# Therapeutic Use of a Selective S1P_1_ Receptor Modulator Ponesimod in Autoimmune Diabetes

**DOI:** 10.1371/journal.pone.0077296

**Published:** 2013-10-24

**Authors:** Sylvaine You, Luca Piali, Chantal Kuhn, Beat Steiner, Virginia Sauvaget, Fabrice Valette, Martine Clozel, Jean-François Bach, Lucienne Chatenoud

**Affiliations:** 1 Université Paris Descartes, Sorbonne Paris Cité, Paris, France; 2 Institut National de la Santé et de la Recherche Médicale, Unité 1013, Paris, France; 3 Actelion Pharmaceuticals Ltd, Allschwil, Switzerland; Children’s Hospital Boston/Harvard Medical School, United States of America

## Abstract

In the present study, we investigated the therapeutic potential of a selective S1P_1_ receptor modulator, ponesimod, to protect and reverse autoimmune diabetes in non-obese diabetic (NOD) mice. Ponesimod was administered orally to NOD mice starting at 6, 10, 13 and 16 weeks of age up to 35 weeks of age or to NOD mice showing recent onset diabetes. Peripheral blood and spleen B and T cell counts were significantly reduced after ponesimod administration. In pancreatic lymph nodes, B lymphocytes were increased and expressed a transitional 1-like phenotype. Chronic oral ponesimod treatment efficiently prevented autoimmune diabetes in 6, 10 and 16 week-old pre-diabetic NOD mice. Treatment withdrawal led to synchronized disease relapse. Ponesimod did not inhibit the differentiation of autoreactive T cells as assessed by adoptive transfer of lymphocytes from treated disease-free NOD mice. In addition, it did not affect the migration, proliferation and activation of transgenic BDC2.5 cells into the target tissue. However, ponesimod inhibited spreading of the T cell responses to islet-specific glucose-6-phosphatase catalytic subunit-related protein (IGRP). Treatment of diabetic NOD mice with ponesimod induced disease remission. However, here again, upon treatment cessation, the disease rapidly recurred. This recurrence was effectively prevented by combination treatment with a CD3 antibody leading to the restoration of self-tolerance. In conclusion, treatment with a selective S1P_1_ modulator in combination with CD3 antibody represents a promising therapeutic approach for the treatment of autoimmune diabetes.

## Introduction

The entry of lymphocytes into lymphoid organs requires different mechanisms implicating chemokines, integrins and selectins. Lymphocyte egress from lymphoid tissues involves the expression of Sphingosine-1-phosphate (S1P) receptors (S1P_1_–S1P_5_), a family of G protein-coupled receptors [1], [2]. These receptors interact with S1P that is a bioactive lysophospholipid present in body fluids and tissues at different concentrations. S1P signaling can mediate diverse cellular responses such as proliferation, cytoskeletal rearrangements, migration and chemotaxis [3]. Therefore, therapeutic strategies targeting this pathway are used to modulate inflammatory processes [4]. The prototype S1P receptor modulator is fingolimod (FTY720) that targets four of the five receptor subtypes. FTY720 inhibits the egress of lymphocytes from thymus and lymph nodes, thereby excluding B and T cells from blood and lymph. Therapeutic efficacy of FTY720 has been extensively demonstrated in models of transplantation [5], [6] and autoimmunity such as experimental autoimmune encephalomyelitis (EAE) [7], systemic lupus erythematosus (SLE) [8], collagen-induced arthritis [9] and colitis [10]. In the non-obese diabetic (NOD) mouse model, that spontaneously develops type 1 diabetes, chronic administration of FTY720 prevented disease and induced diabetes reversal in 50% of treated animals [11], [12]. Translation to the clinical arena showed that FTY720, in association with cyclosporin, was equipotent to mycophenolate mofetil to prevent renal allografts rejection [13]. However, development was stopped due to side-effects, in particular the occurrence of macular edema in a high proportion of patients. Following successful phase II and III trials, fingolimod is now approved for the treatment of relapsing-remitting multiple sclerosis [14].

It has been well established that lymphocyte egress is exclusively mediated by S1P_1_ receptors [15], [16]. Selective S1P_1_ receptor modulators have been developed with the rationale of preserving the immune modulating potential while decreasing side-effects linked to signaling through the other S1P receptors (i.e., smooth muscle cell proliferation and contraction, angiogenesis, vascular permeability). Bolli et al. (Actelion Phamaceuticals Ltd) recently reported the characterization of a potent, orally active, selective S1P_1_ receptor agonist, ponesimod, that is effective at preventing experimental delayed-type hypersensitivity and adjuvant-induced arthritis [17], [18]. Ponesimod is currently in clinical development in multiple sclerosis and psoriasis.

In the present manuscript, we demonstrate the efficacy of ponesimod both in preventing autoimmune diabetes and in reversing established disease in the NOD mouse model. In addition, as rapid disease relapse was invariably observed upon drug withdrawal (a finding also described with FTY720 [12], [13]), and in a clinically oriented view, we propose a combination treatment to overcome this problem. Our results show that a short administration of ponesimod followed by CD3 monoclonal antibody treatment, started a few days before discontinuation of ponesimod, affords long-lasting disease remission.

## Methods

### Mice and Diagnosis of Diabetes

NOD, *IL-4^−/−^* NOD, BDC2.5 NOD and *RAG^−/−^* NOD mice were bred in our animal facility under specific pathogen-free conditions. Glycosuria was measured using colorimetric tests (Glukotest, Roche Diagnostics GmbH) and blood glucose level was measured using ACCU-CHECK Performa glucometer and strips (Roche Diagnostics GmbH). Mice were considered diabetic after two consecutive measurements, made one week apart, showing glycosuria and blood glucose levels >250 mg/dl. Experiments were approved by the Ethic Committee of Paris Descartes University (registered number: P2.CK.153.10).

### Treatment with Ponesimod

Ponesimod was administered orally to NOD mice; the drug was added in the daily food regimen as described [18]. Treatment started at different ages (6, 10, 13 or 16 weeks) and was not discontinued until 35 weeks of age. NOD mice showing recent onset diabetes also received oral ponesimod and disease reversal was monitored by checking for glycosuria 3 times a week.

### Histology

Pancreata were recovered from ponesimod-treated or from age-matched untreated control NOD mice. They were either formaldehyde-fixed or frozen. Five µm thick paraffin sections were stained with hematoxylin/eosin to screen pancreatic islets for the presence of mononuclear cell infiltrates (i.e. insulitis). Frozen sections, fixed in acetone, were incubated for 1 hr with anti-insulin or anti-CD3 primary antibodies. The slides were washed, incubated for 30 min with fluorochrome conjugated secondary antibodies and mounted with Fluoromount G (Southern Biotech) after a final washing step. The confocal images were acquired on a Leica SP5 AOBS and analyzed using ImageJ. Insulitis was scored as follows: no infiltrate = Intact islets, presence of a mononuclear cell infiltrate surrounding the islets = Peri-insulitis, presence of a mononuclear cell infiltrate within the islets = Invasive insulitis). A decrease in insulin staining that confirms the presence of an active β-cell destruction is only seen concomitantly with Invasive insulitis.

### Flow Cytometry

All antibodies used were from BD Biosciences except Foxp3 that was from eBioscience. The class II MHC tetramer carrying a BDC2.5 T cell-specific mimotope (tetAg7/p79) was used as previously described [19]. Briefly, cells were stained with PE-labeled tetramers (5 µg/ml) at 37°C for 3 h. Cell surface antibodies were added during the last 30 min of incubation.

### Adoptive Cell Transfers

Adult *RAG^−/−^* NOD mice were injected i.v. with either total splenocytes (10^7^) or pancreatic lymph node cells (10^6^) recovered from untreated diabetic NOD mice or from 35-week-old diabetes-free NOD mice chronically treated with ponesimod.

### Cytokine Production

Spleen CD4^+^ T cells as well as pancreatic lymph node cells were recovered at different time-points from NOD mice treated or not with ponesimod (beginning of treatment: 6 weeks of age). Cells were stimulated with coated CD3 antibodies (5 µg/ml) and supernatants were recovered after 24, 48 and 72 h of culture. All cytokines were measured by ELISA using DuoSet kit (R&D Systems).

### IFNγ ELISPOT

As previously described [20], PVDF plates (Millipore) were coated with anti-IFNγ capture antibody (U-CyTech). Splenocytes were cultured at 2.5×10^5^/well; lymph nodes cells (5.0×10^4^/well) were co-cultured with 2.0×10^5^ irradiated (35 Gy) splenocytes/well. A mixture of viral peptides (Epstein Barr virus (EBV), cytomegalovirus (CMV) and human immunodeficiency virus (HIV); 7 µM) was used as negative control. The following β-cell autoantigen epitopes were tested: the CD8 T cell epitopes of islet-specific glucose-6-phosphatase catalytic subunit-related protein IGRP_206–14_ and of pro-insulin PI-B_15–23_ and the CD4 T cell epitope of glutamic acid decarboxylase GAD_206–220_ (7 µM). After a 20 hrs culture, IFNγ was detected using biotinylated anti-IFNγ antibody, streptavidin-horseradish peroxidase and Sigma*FAST* NBT-BCIP (Sigma-Aldrich). IFNγ spot readouts were expressed as spot-forming units (SFU)/10^6^ cells.

### Infusion of CFSE-labeled BDC2.5 Cells

Carboxyfluorescein diacetate succinimidyl ester (CFSE)-labeled BDC2.5 T cells were transferred i.v. into 18-wk-old NOD mice (8×10^6^/recipient) that were chronically treated with oral ponesimod since 6 weeks of age. Pancreas, spleen and pancreatic lymph nodes were harvested on day 7 post-infusion. Pancreatic islets were isolated by Histopaque gradient after collagenase P digestion and infiltrating T cells were subsequently collected after trypsin/EDTA treatment.

### *In vivo* Antibody Treatment

CD3-specific antibody (145 2C11) F(ab′)_2_ fragments (the producing cell line was kindly provided by J.A. Bluestone) were produced in house. The treatment consisted in i.v. injections of 50 µg/day for five consecutive days. Diabetic NOD mice, treated with oral ponesimod, entered disease remission. On day 30, CD3 antibody treatment was applied and ponesimod was discontinued 2 days after the last antibody injection.

### Pancreatic Islet Isolation and Transplantation

*RAG^−/−^* NOD pancreatic islets were isolated by density gradient centrifugation (Histopaque, Sigma-Aldrich) after *in situ* digestion with collagenase P (Roche Applied Science) and transplanted (500 islets) under the kidney capsule of diabetic NOD mice. When needed, diabetes was induced by injection of streptozotocin (Sigma-Aldrich) at 225 mg/kg. Diagnosis of graft destruction was made after two sequential glucose measurements >250 mg/dl.

### Statistical Analysis

The occurrence of diabetes was plotted using the Kaplan-Meier method. Statistical comparison between the curves was performed using the logrank (Mantel-Cox) test. When appropriate, the Student’s t test was used. A p value<0.05 was considered statistically significant.

## Results

### 1) Oral Administration of the Selective S1P_1_ Receptor Modulator Ponesimod Protects from Diabetes Development

**Figure 1:**
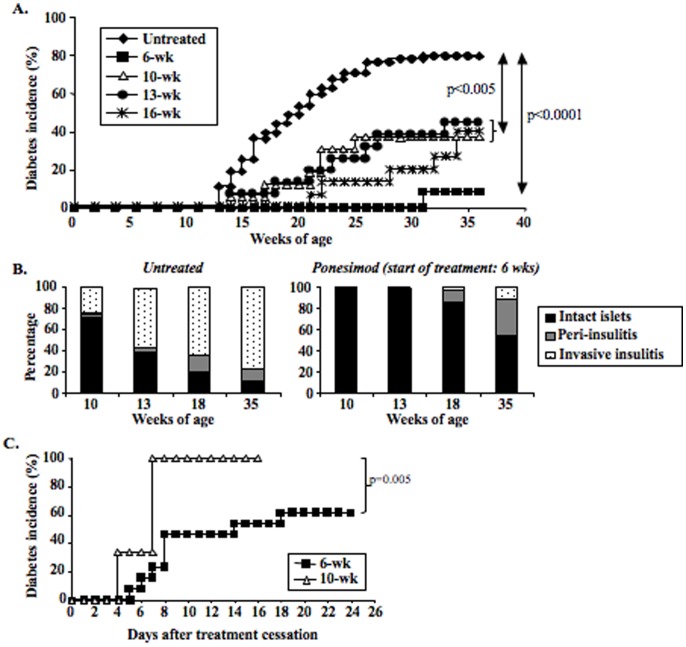
Chronic oral administration of the selective S1P_1_ receptor modulator ponesimod protects from diabetes development. (A) Ponesimod was administered to 6- (n = 23), 10- (n = 16), 13- (n = 16) or 16-week old (n = 15) NOD mice. Treatment was continuous and diabetes occurrence was monitored up to 36 weeks of age. (B) Histological evaluation of insulitis i.e. pancreatic islet infiltration by mononuclear cells. Pancreata were recovered from untreated or ponesimod-treated NOD mice at different time-points (beginning of treatment: 6 weeks of age). Paraffin-embedded pancreas sections were stained with eosin/hematoxylin to score insulitis. (C) Monitoring of diabetes development after withdrawal of ponesimod treatment in 35-week-old diabetes-free NOD mice treated since 6 (n = 13) or 10 (n = 5) weeks of age.

Ponesimod was orally administered (in daily food) to prediabetic NOD mice starting at 6, 10, 13 and 16 weeks of age up to 35 weeks of age (Figure 1A). Significant ponesimod concentrations were detectable in the plasma within 24 hrs of feeding (Table S1). Diabetes development was almost completely abrogated when ponesimod administration was initiated in 6-week-old NOD mice: only 8.9% were diabetic at 35 weeks versus 80% in the control group. Histological analysis showed that islet infiltration was also significantly reduced in ponesimod-treated mice (Figure 1B). At 18 weeks more than 80% of pancreatic islets were intact as compared to less than 20% in controls (Figure 1B). At the end of treatment, only 12% of islets presented an invasive/destructive insulitis as compared to 78% in controls (Figure 1B and Figure S1). In treated mice showing these low insulitis scores, β-cells present within these islets were fully functional as assessed by insulin staining (Figure S1). The effect was less pronounced but still significant when ponesimod treatment was started in 13–16 week-old mice, once invasive/destructive insulitis was established (Figure 1A).

Withdrawal of ponesimod in mice treated from 10 to 35 weeks of age led to a rapid and synchronized relapse of the disease i.e., all mice were diabetic within 7 days of drug discontinuation (Figure 1C). Disease relapse was also observed upon drug withdrawal in mice in which treatment was started at 6 weeks of age but in this case only 60% of the animals relapsed in the first 24 days after discontinuation of ponesimod treatment (Figure 1C).

### 2) Lymphocyte Subsets in Ponesimod-treated Mice

**Figure 2:**
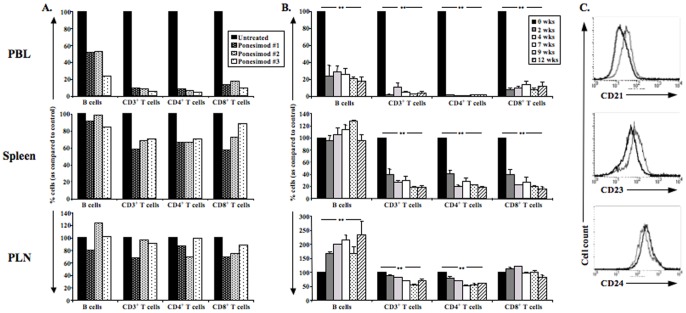
Lymphocyte subsets in peripheral blood and lymphoid organs of ponesimod-treated NOD mice. (A) Six-week-old NOD mice were treated with ponesimod for 24 h and T and B cell proportions were evaluated in peripheral blood (PBL), spleen and pancreatic lymph nodes (PLN) of 3 individual mice. (B) Ponesimod was administered to six-week-old NOD mice. Peripheral blood, spleen and pancreatic lymph nodes were recovered at 2, 4, 7, 9 or 12 weeks to monitor proportions of T cell subsets and B cells (n = 3 for all time-points). Results are expressed as % as compared to untreated age-matched NOD mice (**p<0.005). (C) B cells purified from pancreatic lymph nodes of 18-week-old ponesimod-treated NOD mice (12 weeks of treatment) were stained with antibodies against CD21, CD23 and CD24 (bold line). B cells from age-matched untreated NOD mice were used as controls (thin line).

As soon as 24 hrs after beginning of ponesimod treatment, lymphocytopenia was observed with a 90% decrease in circulating CD3^+^ T cells (both CD4^+^ and CD8^+^) and a 50% decrease in B cells in treated animals as compared to controls (Figure 2A). In the spleen, CD4^+^ and CD8^+^ T cells, but not B cells, were also reduced by 30–40% at 24 hrs after starting drug treatment. At this early time point lymphocyte counts were normal in pancreatic lymph nodes (PLN) (Figure 2A).

After chronic treatment with ponesimod, a marked T cell lymphocytopenia was observed in blood (90–95% reduction) and spleen (70–80% reduction) affecting both CD4^+^ and CD8^+^ T cell subsets (Figure 2B and Figure S2). In PLN, only CD4^+^ T cells were reduced by 50% (Figure 2B and Figure S2).

Proportions of B cells significantly decreased in blood (80% reduction). In PLN, the frequency and the absolute B cell counts doubled after 4 weeks of ponesimod administration (Figure 2B and Figure S2). These B cells, when compared to those recovered from PLN of age-matched untreated NOD mice, showed lower levels of CD21 and CD23 and higher levels of CD24 (Figure 2C), an immature phenotype described for transitional 1 (T1) cells.

**Figure 3:**
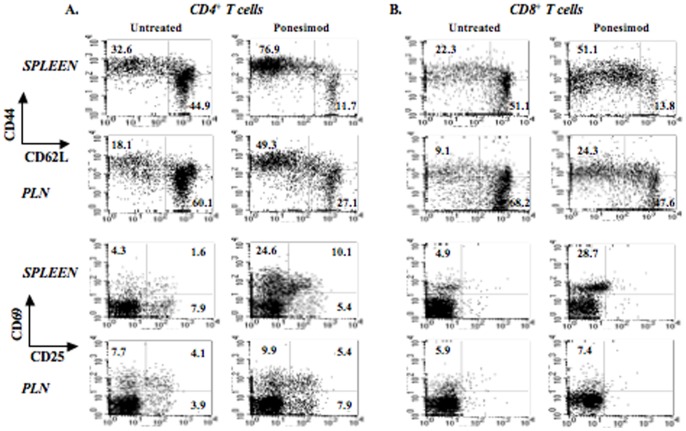
T cells from ponesimod-treated NOD mice exhibit an activated phenotype. Staining with CD44, CD62L, CD25 or CD69 antibodies was performed on spleen or pancreatic lymph node cells recovered from NOD mice treated with ponesimod from 6 to 18 weeks of age. Eighteen-week-old untreated mice were used as controls. (A) Analysis within the CD4^+^ T cell gate. (B) Analysis within the CD8^+^ T cell gate.

T cells recovered from 18-week-old NOD mice treated with ponesimod for 12 weeks showed an activated phenotype. Thus, CD4^+^CD44^high^CD62L^−^ T cells were present in higher proportions in the spleen and PLN of treated mice (76.9% versus 32.6% in the spleen; 49.3% versus 18.1% in PLN) as well as CD8^+^CD44^high^CD62L^−^ T cells (51.1% versus 22.3% in the spleen; 24.3% versus 9.1% in PLN) (Figure 3A and B). Increased expression of CD69 and CD25 was also detected, mainly in the spleen of treated mice (Figure 3A and B). In ponesimod-treated mice, absolute numbers of CD4^+^ Tregs in the spleen decreased (Figure S3A) as a consequence of the decline of CD4^+^ T cells (Figure 2B). Proportions of CD4^+^FoxP3^+^ T cells (Tregs) in the spleen were not significantly modified as compared to untreated controls (i.e., 12.3% versus 12.2% after a 12-week treatment) (Figure S3B). In PLN, a tendency towards an increase in Treg proportions was observed after 12 weeks of treatment (15.5% of total CD4^+^ T cells as compared to 11.2% in age-matched untreated mice) (Figure S3B). We evaluated the *in vitro* suppressive capacity of Tregs isolated from 18-week-old NOD mice treated or not with ponesimod. Tregs from both groups were equally capable of suppressing the proliferation of CD4^+^CD25^−^ T cells when stimulated with CD3 antibodies (Figure S3C).

### 3) Effect of Ponesimod Treatment on Polyclonal Diabetogenic T cells

**Figure 4:**
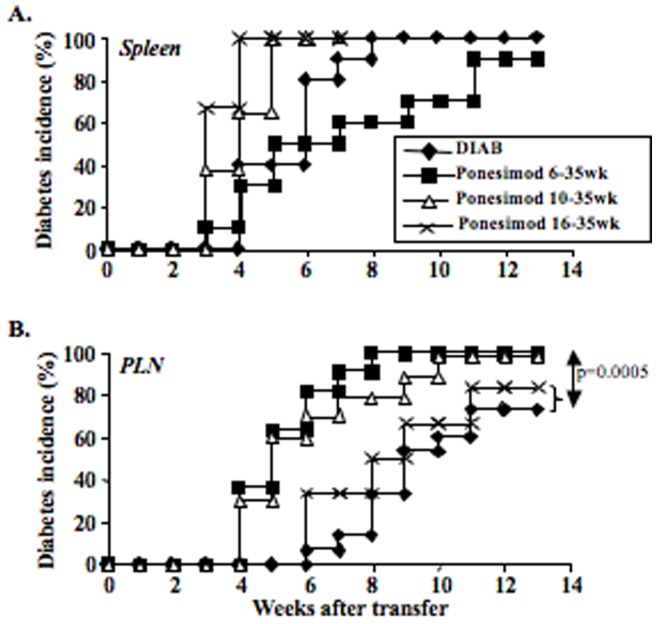
Protected mice still harbor diabetogenic effectors. Adoptive transfer experiments: *RAG^−/−^* NOD mice were injected i.v. with 10^7^ splenocytes (A) or 10^6^ pancreatic lymph nodes cells (B) recovered either from untreated overtly diabetic NOD mice or from 35-week-old diabetes-free ponesimod-treated NOD mice (beginning of treatment at 6, 10 or 16 weeks of age).

To assess the diabetogenic capacity of T cells recovered at the end of ponesimod treatment, adoptive cell transfers into *RAG^−/−^* NOD recipients were performed. Spleen cells from mice, in which treatment with ponesimod was started at 10 or 16 weeks of age, transferred disease as effectively as cells collected from the spleen of diabetic mice (Figure 4A); spleen cells from mice in which treatment was started at 6 weeks of age appeared somewhat less efficient although the difference was not significant. This may be due to a slight reduction in the absolute number of activated CD44^high^CD62L^low^ T cells in the inoculum (i.e. 0.9×10^6^ in ponesimod-treated NOD mice versus 1.2×10^6^ in untreated diabetic NOD mice). In contrast, PLN cells from mice treated from 16 to 35 weeks of age transferred disease as efficiently as PLN cells from diabetic mice (Figure 4B). PLN cells from mice treated at 6 or 10 weeks of age were significantly more efficient to transfer disease than those collected from untreated controls. This may be due to a higher number of activated CD44^high^CD62L^low^ T cells in the inoculum (i.e. 0.22×10^6^ in ponesimod-treated NOD mice versus 0.12×10^6^ in untreated diabetic NOD mice).

**Figure 5:**
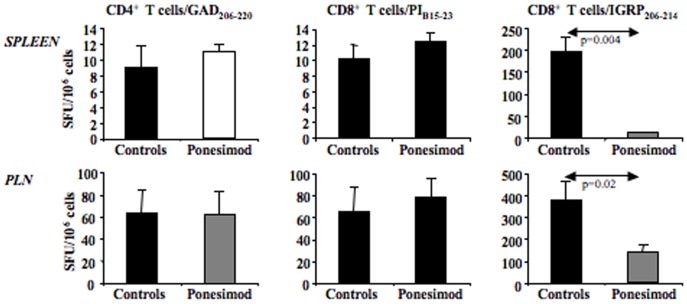
Ponesimod treatment affects autoantigen spreading. Spleen and pancreatic lymph node (PLN) cells were harvested either from control diabetic NOD mice (n = 6) or from 35-week-old diabetes-free NOD mice treated with ponesimod since 6 weeks of age (n = 8). Production of IFNγ was measured by ELISPOT after incubation with PI-B_15–23_, GAD_206–220_ or IGRP_206–214_ peptides. Results are expressed as mean ± SD of spot-forming units (SFU)/10^6^ cells.

We next investigated the effect of ponesimod treatment on β-cell autoantigen-specific CD4^+^ and CD8^+^ T cells by evaluating the IFNγ response to proinsulin (PI-B_15–23_), glutamic acid decarboxylase (GAD_206–220_) or the islet-specific glucose-6-phosphatase catalytic subunit-related protein (IGRP_206–214_). Results showed that a different pattern for IGRP-specific CD8^+^ T cells was observed in ponesimod-treated mice only (Figure 5). Indeed, these IGRP-specific CD8^+^ T cells were almost absent in the spleen and significantly decreased in PLN of treated mice in contrast to autoreactive PI-B_15–23_-specific CD8^+^ T cells and GAD_206–214_-specific CD4^+^ and (Figure 5).

**Figure 6:**
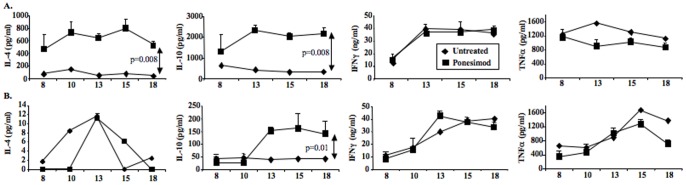
Cytokine production. Spleen CD4^+^ T cells (A) or total pancreatic lymph node cells (B) were isolated from age-matched NOD mice orally treated or not with ponesimod. Administration started at 6 weeks of age. IL-4, IL-10, IFNγ and TNFα production was measured by ELISA after an *in vitro* 48 h stimulation with CD3 antibodies (5 µg/ml).

Results showed that following polyclonal stimulation spleen cells produced higher amounts of IL-4 and IL-10 as compared to those from age-matched untreated NOD mice, a pattern that was sustained with time (Figure 6A). Pancreatic lymph nodes of NOD mice treated with ponesimod also secreted large amounts of IL-10 but not of IL-4 (Figure 6B). No significant change was observed for IFNγ and TNFα. To further assess the role of Th2 cells in the protective effect of ponesimod, we treated NOD mice deficient for IL-4 (IL-4^−/−^). Results showed that in these mice ponesimod treatment prevented diabetes development as efficiently as in wild-type NOD mice (Figure S4).

### 4) Ponesimod does not Abrogate Homing, Activation and Proliferation of T cell Receptor (TCR) Transgenic Autoreactive T cells

**Figure 7:**
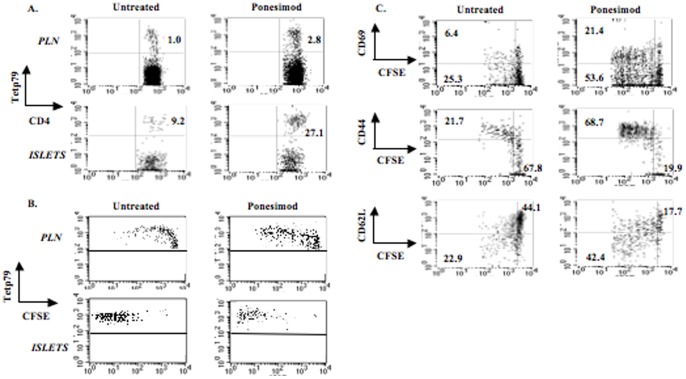
Migration and proliferation of CD4^+^BDC2.5 T cells in pancreatic tissues and lymph nodes of ponesimod-treated NOD mice. Six-week-old NOD mice were treated with oral ponesimod for 12 consecutive weeks. Eight million CFSE-labeled CD4^+^BDC2.5^+^ T cells were then infused in the treated mice. Recipients were sacrificed on day 7 post-transfer and cell suspensions were recovered from pancreatic islets and lymph nodes and analyzed by flow cytometry. (A) Tet/p79 tetramer staining (MHC class II tetramer carrying a BDC2.5 T cell-specific peptide p79) in the CD4^+^ T cell gate. (B) Proliferation of BDC2.5 T cells measured by CFSE staining in the CD4^+^Tet/p79^+^ T cell gate. (C) Expression of CD69, CD44 and CD62L by proliferating BDC2.5 T cells (CD4^+^Tet/p79^+^ T cell gate). The results shown are representative of three independent experiments.

The rapid relapse of diabetes observed after withdrawal of ponesimod as well as the diabetogenic capacity of spleen and pancreatic lymph node cells suggested that β-cell antigen-specific T lymphocytes are still primed and expand during treatment. To further explore this issue, we infused CFSE-labeled CD4^+^BDC2.5 transgenic T cells into 18 week-old NOD mice that had received oral ponesimod since 6 weeks of age. To specifically detect CD4^+^BDC2.5 T cells, we used I-A^g7^ MHC class II tetramers (tetp79) presenting a synthetic peptide 1040–79 (p79) selectively recognized by the BDC2.5 T cells [19]. As in control animals, BDC2.5 cells preferentially migrated to the target tissue due to the presence of the cognate antigen. In untreated NOD mice, transgenic T cells represented 1% and 9.2% of total CD4^+^ T cells in PLN and within the islet infiltrates, respectively (Figure 7A). This proportion was increased three-fold in ponesimod-treated mice, reaching 2.8% and 27.1% in the respective organs. BDC2.5 cells proliferated very actively: in PLN, almost 60% of them divided more than three times (as compared to 12.7% in untreated mice) and the vast majority of islet-infiltrating BDC2.5 T cells underwent more than 5 divisions (Figure 7B). Proliferating cells expressed CD69 and high levels of CD44 while CD62L staining decreased (Figure 7C).

### 5) Treatment of Established Diabetes

**Figure 8:**
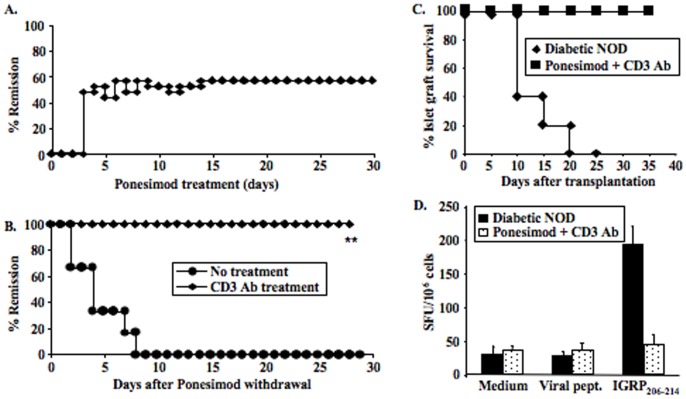
Oral administration of ponesimod can reverse established diabetes and combination with CD3-specific antibodies restores self-tolerance. (A) Recently diagnosed diabetic NOD mice were treated with oral ponesimod (n = 23) and diabetes remission was evaluated by glycosuria and glycemia measurements. Durable remission occurred in 57% of mice. (B) Administration of CD3-specific monoclonal antibodies (50 µg/day i.v. for 5 consecutive days) was performed in NOD mice that showed remission upon continuous ponesimod treatment (n = 6). Oral ponesimod was withdrawn at the end of CD3 antibody treatment. All mice that received ponesimod and CD3 antibody treatment showed durable disease remission. In contrast, all mice treated with ponesimod alone showed disease relapse by 1 week following ponesimod withdrawal (**p = 0.003). (C) NOD mice showing sustained diabetes remission after combined treatment with ponesimod and CD3-specific antibodies were treated with streptozotocin (225 mg/kg) to destroy all endogenous β-cells and artificially induce diabetes. Three days later, they were transplanted under the kidney capsule with 500 syngeneic pancreatic islets isolated from *RAG^−/−^* NOD mice (n = 3). Untreated overtly diabetic NOD mice were used as controls (n = 3). Survival of syngeneic islets was significantly prolonged in the ponesimod/CD3 antibody-treated NOD mice as compared to untreated NOD recipients. (D) Spleen cells were harvested from NOD mice having received combined treatment with ponesimod and CD3 antibodies and transplanted with syngeneic islets. Production of IFNγ was measured at the time of rejection by ELISPOT after a 20 hrs-incubation of total splenocytes with IGRP_206–214_ peptides or control viral peptides (EBV, CMV, HIV). Results are expressed as mean ± SD of spot-forming units (SFU)/10^6^ cells and represent the average response of 3 mice/group.

We investigated whether continuous oral administration of ponesimod could reverse autoimmune diabetes, once hyperglycemia appeared. Diabetic NOD mice were treated within the first 2 days of hyperglycemia and 60% of them (13/23) entered stable remission (Figure 8A). However, upon cessation of ponesimod administration, disease relapsed in all mice within 10 days, an observation that is consistent with results described above showing an accumulation of autoreative T effector cells (BDC2.5 transgenic T cells) in pancreatic lymph nodes and within islets.

To reduce the relapse rate, we combined ponesimod with a CD3-specific monoclonal antibody. Indeed, we previously established that treatment with CD3 antibody induces diabetes remission in overtly diabetic NOD mice by restoring self-tolerance [21], [22]. Thus, diabetic NOD mice, that had entered remission following continuous oral administration of ponesimod for 30 days, received 5 injections of CD3 antibody F(ab′)_2_ fragments (50 µg/day). Two days after the last CD3 antibody injection, on day 37, ponesimod treatment was withdrawn. All mice treated with CD3 antibody remained normoglycemic while untreated animals developed diabetes within 2 to 8 days following cessation of ponesimod treatment (Figure 8B).

To monitor the β-cell-specific autoreactivity in protected mice, we performed syngeneic pancreatic islets grafts. NOD mice in remission 50 days after the last CD3 antibody injection were treated with streptozotocin to destroy endogenous β-cells. Five hundred islets recovered from *RAG* ^−/−^ NOD donors were transplanted under the kidney capsule. Syngeneic islets were destroyed within 7 to 10 days in untreated diabetic NOD recipients due to relapse of autoimmunity (Figure 8C). In contrast, they survived long-term in recipients previously treated with ponesimod and CD3 antibodies (Figure 8C). In mice, the proportion of IGRP-specific CD8^+^ autoreactive T cells in the spleen was very low when compared to that observed in untreated diabetic NOD controls (Figure 8D).

## Discussion

In this study, we demonstrated the therapeutic effect in experimental autoimmune diabetes of ponesimod, a recently characterized molecule delivered orally and which blocks lymphocyte egress by selectively targeting S1P_1_ receptors. Our major findings are that disease prevention or reversal was obtained following chronic ponesimod administration but rapid relapse was observed upon treatment cessation. To overcome this problem, a short-term combination treatment associating in a consecutive fashion ponesimod with CD3 antibody induced long-lasting remission of diabetes thus providing a cure for the disease.

An effective protection from disease in 90% of mice at 35 weeks of age was observed when ponesimod treatment was applied chronically starting in young 6 week-old prediabetic mice. If treatment start was delayed to 10 to 16 weeks of age, the effect was still significant with a protection observed in about 60% of animals. At the time of established hyperglycemia, chronic ponesimod treatment induced effective remission with a stable return to normal metabolic control.

As already described for fingolimod, we observed that, at treatment cessation, disease relapse occurred with two interesting characteristics. First, the later the treatment was started, the larger the proportion of relapsing animals was. Thus, in our study 100% disease relapse was observed when treatment was started at 10 weeks of age as compared to 60% when treatment was started at 6 weeks of age. These figures are fully concordant with those observed with FTY720 [11], [12], [23]. In mice in which FTY720 treatment was started as early as 4 weeks of age and was protracted for 35 weeks, Maki et al. reported a relapse in only 33% of animals [11]. Secondly, independent of the proportion of disease relapse observed at treatment cessation, its kinetics was synchronized and nearly all mice became diabetic within 7 to 10 days following drug withdrawal.

The relapse and its synchronization are well explained by the fact that the primary effect of ponesimod is to sequester B and T cells in the thymus and lymphoid organs where they can be primed by cognate antigens but cannot egress. Our data provides evidence that activation of autoreactive T cells is not inhibited by ponesimod, with however, an interesting reservation that we shall discuss below. In fact, analysis of T cell subsets in the spleen and PLN collected from mice treated chronically with ponesimod, consistently showed an increase in CD4^+^ and CD8^+^ T cells presenting an activated/memory phenotype i.e. CD44^high^CD62L^−^, the subset known to include activated pathogenic T cells [24], [25]. These data are well in keeping with those of the adoptive transfer experiments where both spleen and pancreatic lymph node cells from treated mice had a fully preserved and sometimes greater capacity to induce disease into immunoincompetent recipients. As expected, this capacity correlated with the numbers of activated pathogenic cells present within the cell inocula. Addressing more precisely the homing of autoreactive effectors to the islets and draining lymph nodes, T cell receptor transgenic BDC 2.5 cells, specific for chromogranin A [26], rapidly migrated to the target tissue where they also expanded when injected into ponesimod-treated recipients. These results extend the data obtained by Penaranda et al. indicating that starting FTY720 treatment following the transfer of CFSE-labeled BDC2.5 T cells did not prevent their expansion in the pancreas [23]. However, in these experiments, treatment was started after the transfer, and therefore no direct indication was obtained on a potential influence of the drug on the homing of autoreactive effectors. In addition, our results also suggest that ponesimod did not have a major apoptotic effect on the transferred autoreactive effectors.

We also analyzed autoreactive CD4^+^ and CD8^+^ T cells using an IFNγ ELISpot test [20]. Results showed that when ponesimod administration was started at 6 weeks of age, the chronic treatment severely impaired the IGRP-specific CD8^+^ T cell response. This data suggest that although ponesimod does not globally affect the activation and expansion of diabetogenic effectors it may, if administered early enough, interfere with antigen spreading. Antigen/epitope spreading - the progressive diversification of the autoimmune response from one epitope to another expressed by the same or by a distinct autoantigen (i.e., intramolecular spreading versus intermolecular spreading)- is in fact the major driver underlying the chronicity of the autoimmune responses. It was initially described in EAE and then extended to demyelinating diseases that follow viral infections (e.g., Theiler’s murine encephalomyelitis) as well as autoimmune diabetes [27]–[30]. Concordant data show that the IGRP response lies downstream in the anti-β-cell response with insulin/proinsulin representing, at least in the NOD mouse, the putative primary autoantigen [31], [32].

An increased and sustained ability of T cells to produce IL-4 and IL-10 was shown in ponesimod-treated NOD mice as compared to controls. Ponesimod treatment was equally effective in IL-4 invalidated (IL-4^−/−^) and wild-type NOD mice. The role of IL-10 needs further investigation as this cytokine may promote and be produced by tolerogenic dendritic cells, by regulatory type 1 (Tr1) T cells [33], [34] and also by regulatory B cells [35]–[37] including transitional B cells. This latter subset was detected in increasing proportions in PLN of prediabetic mice treated chronically with ponesimod.

Concerning clinical translation in autoimmune diabetes of ponesimod, all data are consistent in showing high efficacy in both prevention and treatment of established disease when the drug is administered chronically. A major problem, which has not been observed in EAE or in multiple sclerosis, is the rapid recurrence of disease after discontinuation of treatment.

Our approach to this problem has been to use combination therapy with CD3 antibodies that had shown effectiveness to restore self-tolerance in the mouse and the clinic in established autoimmune diabetes [38]–[41]. CD3-specific monoclonal antibodies promote short-term immunosuppression and, when adequately delivered, they induce immune tolerance, an effect shown for both autoantigens [21], [42] and alloantigens [43]. The biological effects observed during treatment are the antigenic modulation of the CD3/TCR complex at the T cell surface and a partial depletion of T cells through redirected lysis and apoptosis [21]. Interestingly, this apoptosis preferentially targets antigen-specific pathogenic T cells while it spares Tregs [43]–[45]. This explains, at least in part, that following treatment, immune reconstitution occurs in a local environment which favors the expansion of antigen-specific Tregs and the maintenance over long term of the immune tolerance induced [21], [38], [42], [46].

When applied at the end of ponesimod treatment, a short CD3 antibody course completely prevented disease rebound by restoring self-tolerance as well demonstrated by the present syngeneic transplant data. Syngeneic islets are rapidly destroyed when implanted into untreated diabetic recipients or into mice weaned from ponesimod showing disease relapse, well reflecting the ongoing uncontrolled autoimmune response. At variance, syngeneic islets survive long-term in mice that received CD3 antibody following ponesimod treatment as witnesses of the existing *in vivo* operational immune tolerance. Further supporting the capacity of CD3 antibody treatment to control pathogenic responses, are the *ex vivo* results showing the major decrease of the number of IGRP-specific CD8^+^ lymphocytes in these same mice.

This type of combination therapy is interesting as the two products have a complementary mode of action. On one hand, ponesimod allows rapid action on pathogenic lymphocytes that are ‘excluded’ from any proximity to the target organ. Moreover, it is a compound that is delivered orally, for which the clinical data on tolerability indicate a good safety [47]. Importantly, the half-life of ponesimod is significantly shorter than that of fingolimod (30 hrs versus 8 days, respectively) allowing rapid reversibility of its effect following discontinuation of treatment [47], [48]. Like fingolimod, ponesimod showed acceptable low risk of infections in multiple sclerosis patients ([14], [49], Freedman et al. personal communication). Given its strict specificity for lymphocyte S1P_1_ receptor, ponesimod, at variance with fingolimod, does not elicit pro-fibrotic responses linked to recognition of S1P_2_ and S1P_3_ receptors [50]. On the other hand, CD3 antibodies combine a short immunosuppressive action, given the limited window of administration, with a unique tolerance promoting ability that, as shown by our data, is not counteracted by the ponesimod treatment. It is our working hypothesis that the predominant effect of CD3 antibodies on activated T cells, as demonstrated in autoimmunity and more recently in transplantation [21], [43], associated to the capacity of ponesimod to trap autoreactive T cells within lymph nodes without preventing their activation may constitute the basis for further complementarity of action of these two drugs. This also well explains both the efficacy of the combination and the protection from relapse of the disease when ponesimod treatment is suspended.

The significant number of immune intervention trials conducted in type 1 diabetes indicates that single-drug approaches will not arrest disease progression in a satisfactory way. Combination therapies will be needed. Given the presently ongoing pharmaceutical development of the two drugs proposed here, we would like to suggest considering proof of concept combination trials in type 1 diabetes in a not too distant future.

## Supporting Information

Figure S1 **Monitoring of insulitis.**
(PPT)

Figure S2 **Lymphocyte counts in lymphoid organs of ponesimod-treated NOD mice.**
(PPT)

Figure S3 **Foxp3^+^ Tregs are less sensitive than conventional T cells to ponesimod-induced lymphopenia in pancreatic lymph nodes (PLN).**
(PPT)

Figure S4 **The protective effect of ponesimod is not IL-4-dependent.**
(PPT)

Table S1 **Ponesimod concentrations measured in the serum of treated NOD mice.**
(DOC)
