# Supplementary material for: Therapeutic Use of a Selective S1P1 Receptor Modulator Ponesimod in Autoimmune Diabetes
Source: PLoS One. 2013 Oct 24;8(10):e77296. doi: 10.1371/journal.pone.0077296 (PMC3811978; doi:10.1371/journal.pone.0077296)
Supplement: Figure S1 — Monitoring of insulitis. (PPT) [file pone.0077296.s001.ppt]

## Slide 1
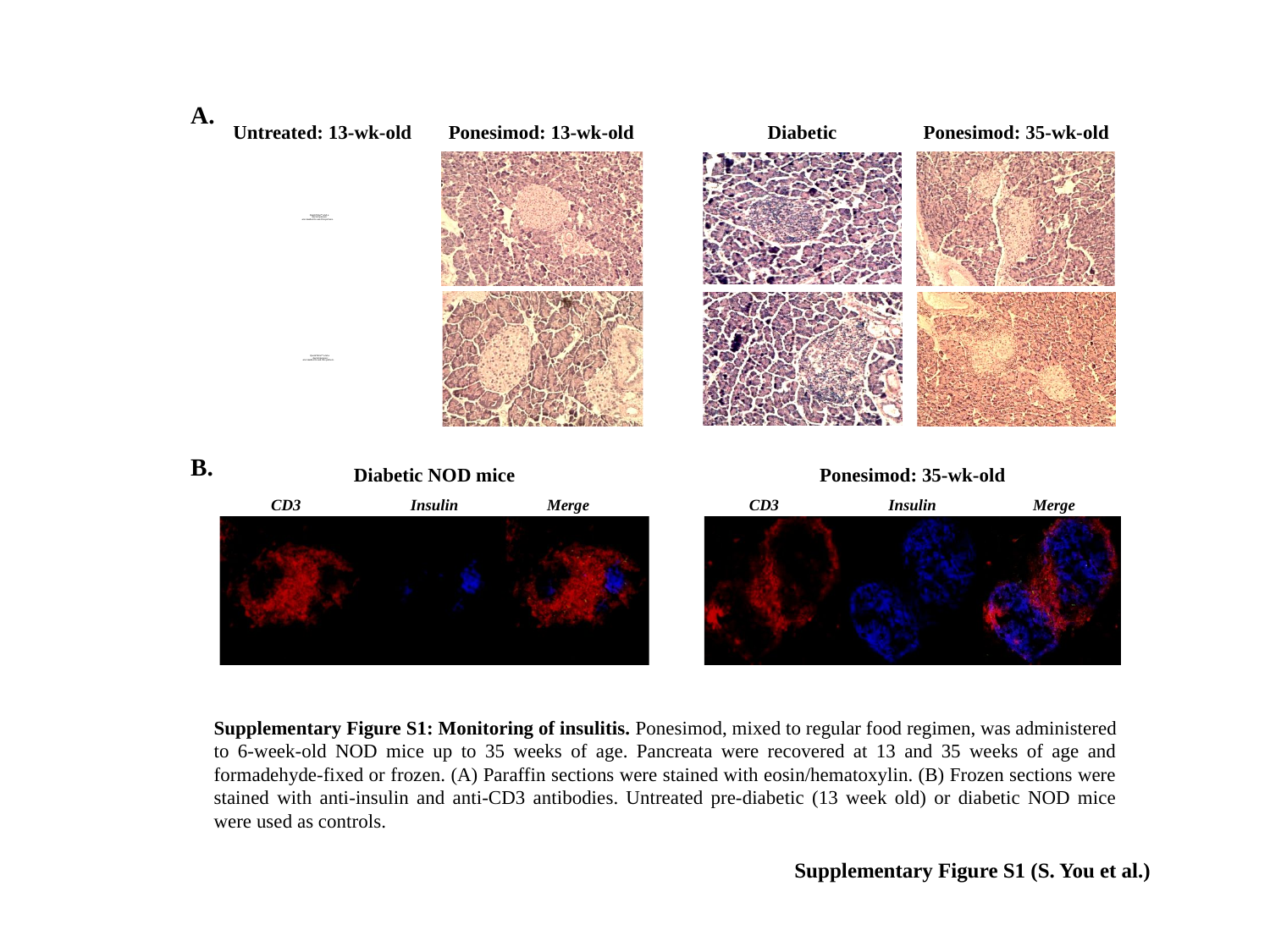

A.
Untreated: 13-wk-old
Ponesimod: 13-wk-old
Diabetic
Ponesimod: 35-wk-old
B.
Diabetic NOD mice
Ponesimod: 35-wk-old
CD3
Insulin
Merge
CD3
Insulin
Merge
Supplementary Figure S1: Monitoring of insulitis. Ponesimod, mixed to regular food regimen, was administered to 6-week-old NOD mice up to 35 weeks of age. Pancreata were recovered at 13 and 35 weeks of age and formadehyde-fixed or frozen. (A) Paraffin sections were stained with eosin/hematoxylin. (B) Frozen sections were stained with anti-insulin and anti-CD3 antibodies. Untreated pre-diabetic (13 week old) or diabetic NOD mice were used as controls.
Supplementary Figure S1 (S. You et al.)
