# Supplementary material for: Therapeutic Use of a Selective S1P1 Receptor Modulator Ponesimod in Autoimmune Diabetes
Source: PLoS One. 2013 Oct 24;8(10):e77296. doi: 10.1371/journal.pone.0077296 (PMC3811978; doi:10.1371/journal.pone.0077296)
Supplement: Figure S2 — Lymphocyte counts in lymphoid organs of ponesimod-treated NOD mice. (PPT) [file pone.0077296.s002.ppt]

## Slide 1
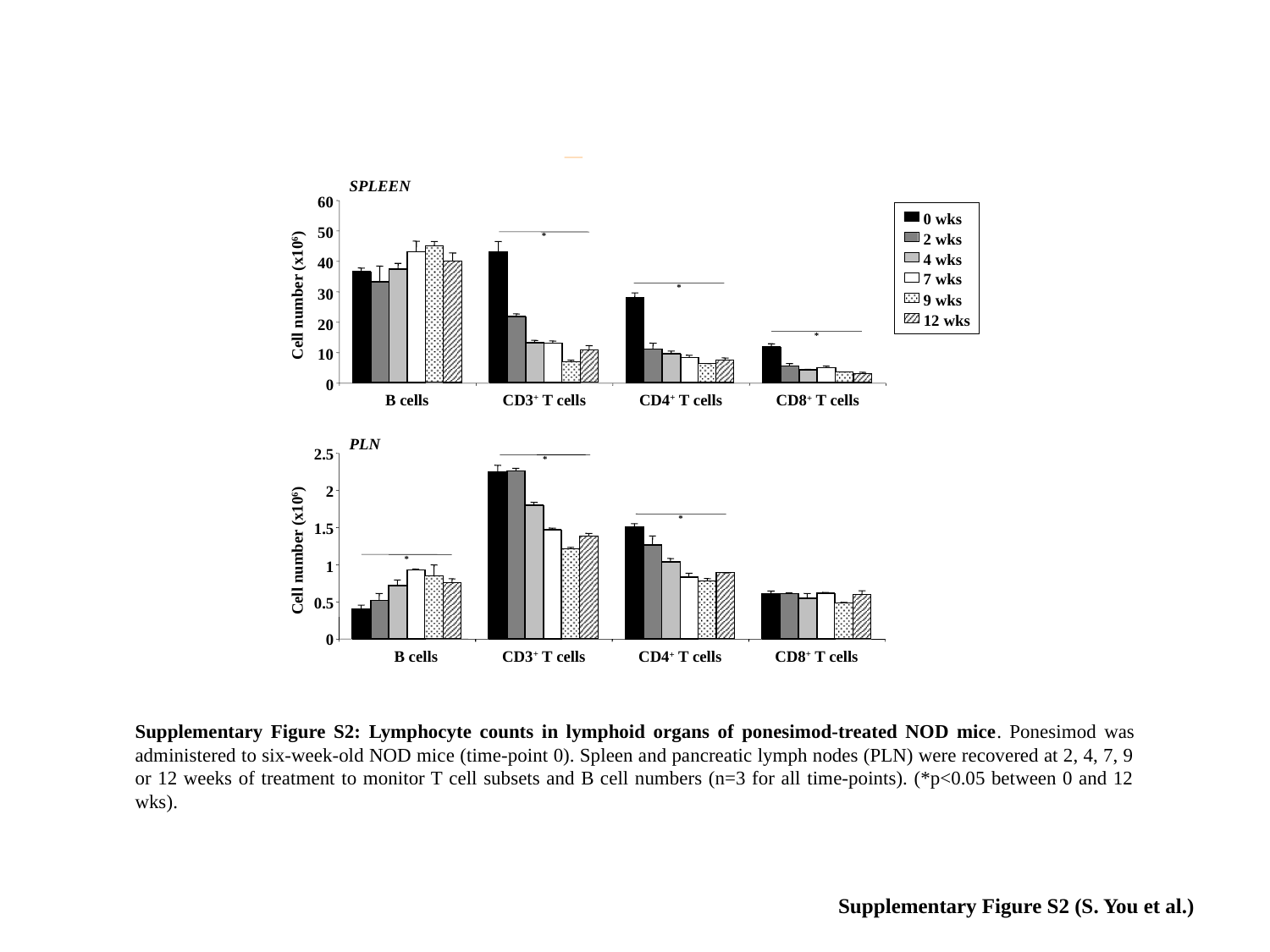

SPLEEN
60
0 wks
2 wks
4 wks
7 wks
9 wks
12 wks
*
50
40
*
30
Cell number (x106)
*
20
10
0
B cells
CD3+ T cells
CD4+ T cells
CD8+ T cells
PLN
*
2.5
2
*
1.5
*
Cell number (x106)
1
0.5
0
CD8+ T cells
B cells
CD3+ T cells
CD4+ T cells
Supplementary Figure S2: Lymphocyte counts in lymphoid organs of ponesimod-treated NOD mice. Ponesimod was administered to six-week-old NOD mice (time-point 0). Spleen and pancreatic lymph nodes (PLN) were recovered at 2, 4, 7, 9 or 12 weeks of treatment to monitor T cell subsets and B cell numbers (n=3 for all time-points). (*p<0.05 between 0 and 12 wks).
Supplementary Figure S2 (S. You et al.)
