# Supplementary material for: Therapeutic Use of a Selective S1P1 Receptor Modulator Ponesimod in Autoimmune Diabetes
Source: PLoS One. 2013 Oct 24;8(10):e77296. doi: 10.1371/journal.pone.0077296 (PMC3811978; doi:10.1371/journal.pone.0077296)
Supplement: Figure S3 — Foxp3+ Tregs are less sensitive than conventional T cells to ponesimod-induced lymphopenia in pancreatic lymph nodes (PLN). (PPT) [file pone.0077296.s003.ppt]

## Slide 1
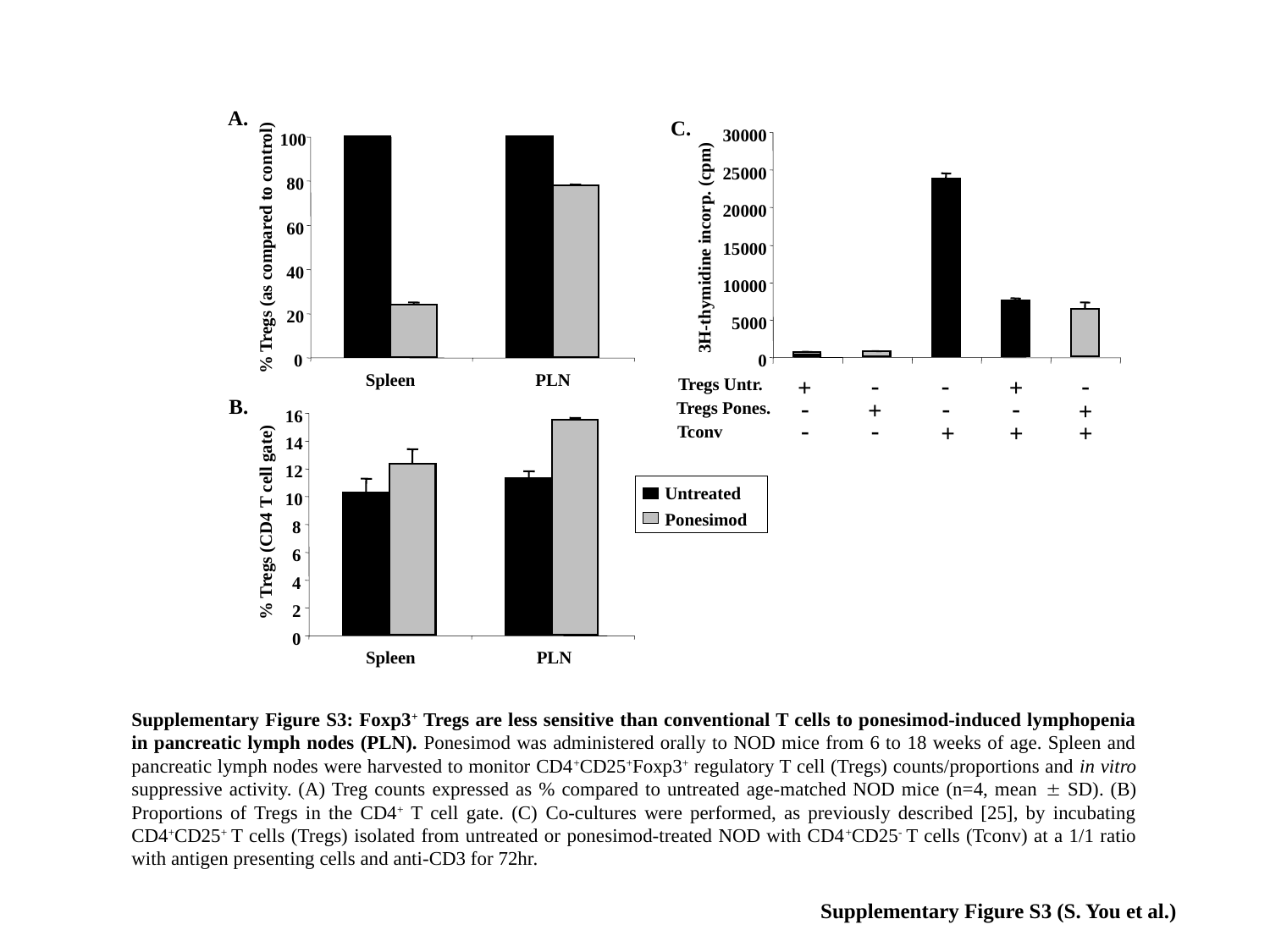

A.
C.
30000
100
25000
80
20000
60
3H-thymidine incorp. (cpm)
% Tregs (as compared to control)
15000
40
10000
20
5000
0
0
-
-
-
+
+
Spleen
PLN
Tregs Untr.
-
-
-
B.
+
+
Tregs Pones.
16
-
-
+
+
+
Tconv
14
12
Untreated
Ponesimod
10
% Tregs (CD4 T cell gate)
8
6
4
2
0
Spleen
PLN
Supplementary Figure S3: Foxp3+ Tregs are less sensitive than conventional T cells to ponesimod-induced lymphopenia in pancreatic lymph nodes (PLN). Ponesimod was administered orally to NOD mice from 6 to 18 weeks of age. Spleen and pancreatic lymph nodes were harvested to monitor CD4+CD25+Foxp3+ regulatory T cell (Tregs) counts/proportions and in vitro suppressive activity. (A) Treg counts expressed as % compared to untreated age-matched NOD mice (n=4, mean ± SD). (B) Proportions of Tregs in the CD4+ T cell gate. (C) Co-cultures were performed, as previously described [25], by incubating CD4+CD25+ T cells (Tregs) isolated from untreated or ponesimod-treated NOD with CD4+CD25- T cells (Tconv) at a 1/1 ratio with antigen presenting cells and anti-CD3 for 72hr.
Supplementary Figure S3 (S. You et al.)
