# Supplementary material for: Therapeutic Use of a Selective S1P1 Receptor Modulator Ponesimod in Autoimmune Diabetes
Source: PLoS One. 2013 Oct 24;8(10):e77296. doi: 10.1371/journal.pone.0077296 (PMC3811978; doi:10.1371/journal.pone.0077296)
Supplement: Figure S4 — The protective effect of ponesimod is not IL-4-dependent. (PPT) [file pone.0077296.s004.ppt]

## Slide 1
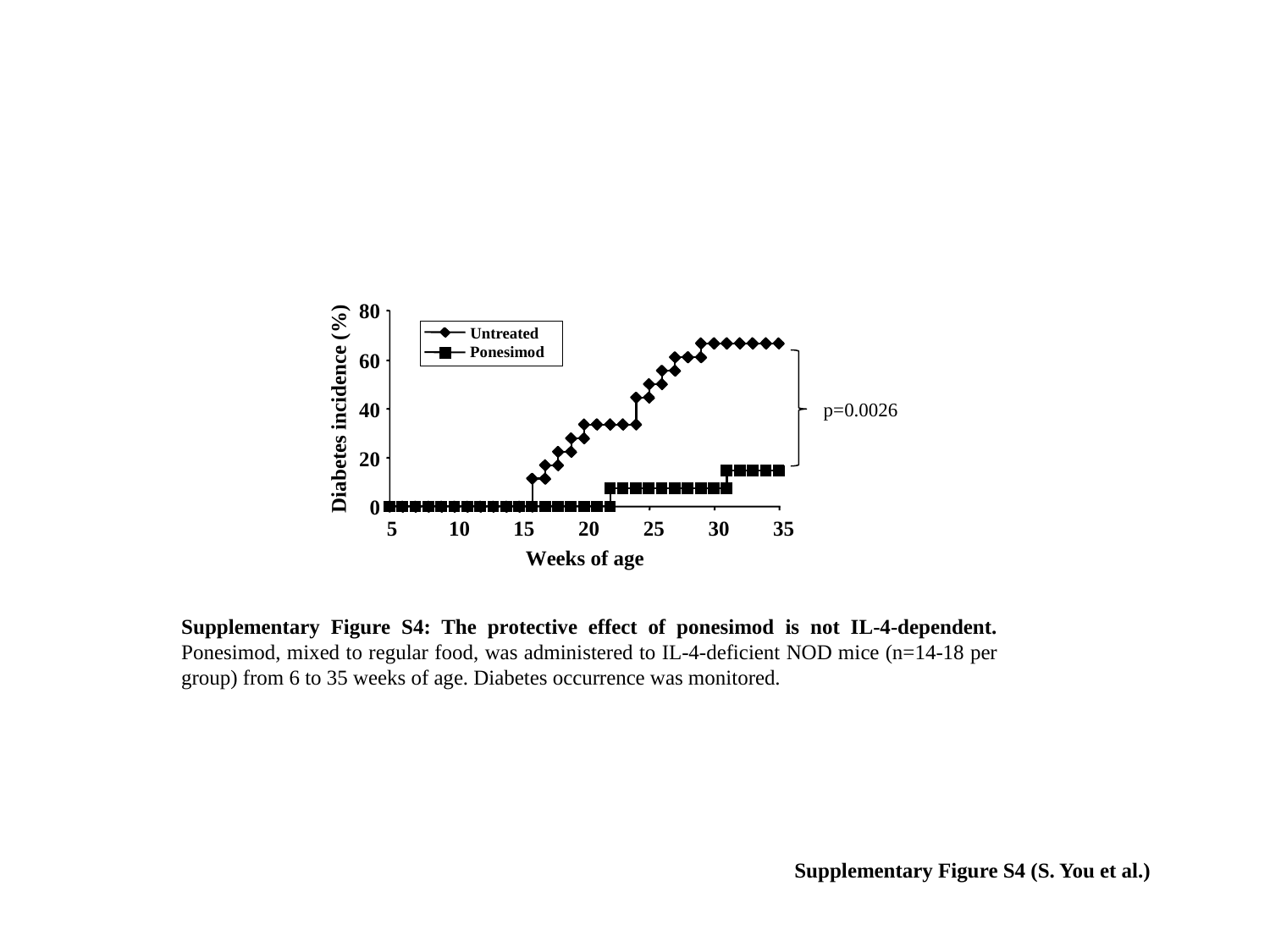

80
Untreated
Ponesimod
60
p=0.0026
Diabetes incidence (%)
40
20
0
5
10
15
20
25
30
35
Weeks of age
Supplementary Figure S4: The protective effect of ponesimod is not IL-4-dependent. Ponesimod, mixed to regular food, was administered to IL-4-deficient NOD mice (n=14-18 per group) from 6 to 35 weeks of age. Diabetes occurrence was monitored.
Supplementary Figure S4 (S. You et al.)
