# Supplementary material for: Therapeutic Use of a Selective S1P1 Receptor Modulator Ponesimod in Autoimmune Diabetes
Source: PLoS One. 2013 Oct 24;8(10):e77296. doi: 10.1371/journal.pone.0077296 (PMC3811978; doi:10.1371/journal.pone.0077296)
Supplement: Table S1 — Ponesimod concentrations measured in the serum of treated NOD mice. (DOC) [file pone.0077296.s005.doc]

**Supplementary Table S1:** Ponesimod concentrations measured in the serum of treated NOD mice

| **Mice/treatment** | **Ponesimod conc (ng/ml)** |
| --- | --- |
| Control NOD, 8-wk  Control NOD, 8-wk  NOD 8-wk, treated with Ponesimod for 24h  NOD 8-wk, treated with Ponesimod for 24h  NOD 8-wk, treated with Ponesimod for 24h  NOD 8-wk, treated with Ponesimod for 48h  NOD 8-wk, treated with Ponesimod for 48h  NOD 8-wk, treated with Ponesimod for 48h | 0  0  1130  2049  2190  3120  2470  1808 |
| Control NOD, 13-wk  NOD 13-wk, treated with Ponesimod for 7 weeks  NOD 13-wk, treated with Ponesimod for 7 weeks  NOD 13-wk, treated with Ponesimod for 7 weeks | 0  1790  1150  681 |
| Control NOD, 16-wk  NOD 16-wk, treated with Ponesimod for 10 weeks  NOD 16-wk, treated with Ponesimod for 10 weeks | 0  1960  1960 |
| Diabetic NOD  Diabetic NOD treated with Ponesimod for 6 weeks: in remission  Diabetic NOD treated with Ponesimod for 6 weeks: in remission | 0  607  1309 |
